# Clinical Implementation and Initial Validation of Respiratory-Gated Stereotactic Body Radiotherapy for Thoracoabdominal Tumors Under Abdominal Compression Using an Anzai Laser-Based Gating Device With Visual Guidance on an Elekta Linear Accelerator

**DOI:** 10.7759/cureus.59638

**Published:** 2024-05-04

**Authors:** Masataka Hoshina, Masaya Noguchi, Masato Takanashi, Kouichi Masuda, Shinji Sugahara

**Affiliations:** 1 Department of Radiology and Radiation Oncology, Tokyo Medical University Ibaraki Medical Center, Inashiki-gun, JPN

**Keywords:** vmat, imrt, lung tumor, sabr, sbrt, stereotactic body radiotherapy, laser sensor, visual guidance, abdominal compression, respiratory gating

## Abstract

We have clinically implemented gated stereotactic body radiotherapy under abdominal compression using an Anzai laser-based gating device with visual guidance in combination with an Elekta linear accelerator. To ensure accuracy, we configured the gating window for each patient by correlating the respiratory curve from the laser sensor and the tumor positions from the 4D computed tomography (CT) images reconstructed with the aid of the respiratory curve. This allowed us to define a patient-specific gating window to keep the tumor displacement below 5 mm from the end-expiration, assuming the reproducibility of the tumor trajectories and the laser-based body surface measurements. Results are summarized as follows: 1) A patient-specific gating window internal target volume (ITV) with a prespecified maximum tumor displacement relative to the end-expiration was obtained by acquiring a 4D CT consisting of 20 phase CT sets and a respiratory curve from the Anzai system. 2) Respiratory hysteresis was managed by setting two different thresholds on the respiratory curve based on the predetermined maximum tumor displacement relative to end-expiration. 3) Abdominal compression increased gating window width, thereby presumably leading to faster gated-beam delivery. 4) Gamma index pass rates in sliding-window gated intensity-modulated radiotherapy (IMRT) were superior to those in gated volumetric modulated arc therapy (VMAT). 5) Intrafraction gated cone-beam computed tomography (CBCT) demonstrated that the tumor appeared to remain within the gating window ITV during the stereotactic gated sliding-window IMRT. In conclusion, we have successfully implemented gated stereotactic body radiotherapy at our clinic and achieved a favorable clinical validation result. More cases need to be evaluated to increase the validity.

## Introduction

Respiratory-gated radiotherapy was first proposed in 1989 by Ohara at the University of Tsukuba [1]. Until the mid-1990s, it was only used by this institution [2,3]. According to a recent survey report by the American Association of Physicists in Medicine (AAPM) Task Group (TG) 324, out of 536 institutions, 60% used the internal target volume (ITV) method, 14% used breath hold, 11% used abdominal compression, and 10% used gating [4]. The ITV method is a standard motion management technique, where a treatment volume is defined by the envelope of the lesion delineated on all phases of the 4D CT. The AAPM TG 76 report, which is nearly identical to the AAPM 91 report, recommends considering respiratory management techniques if a range of motion greater than 5 mm in any direction is observed [3,5]. In other words, if abdominal compression fails to achieve the 5 mm range of motion, a gating technique may need to be considered. Respiratory gating aims to reduce doses to organs at risk without impairing doses to the tumor. A normal tissue complication probability (NTCP) model estimated that respiratory gating reduced pneumonitis risk from 43% to 32% for the highest-risk lung cancer patients [6]. Previous gating techniques have included free breathing gating with patient body surface measurements, or with fluoroscopic imaging of metal markers embedded near the tumor as a surrogate [1,7-10]. Respiratory gating with audio guidance and visual feedback was also reported to improve the reproducibility and regularity of respiratory amplitude and period [8]. To the authors' knowledge, no studies have reported respiratory gating under abdominal compression on a standard linear accelerator (linac).

The purpose of this study was to clinically implement and validate our respiratory-gated stereotactic radiotherapy technique under abdominal compression using an Anzai laser-based gating device with visual guidance in combination with an Elekta linac.

## Technical report

We started respiratory-gated radiotherapy using an Elekta linac, Axesse (Elekta AB, Stockholm, Sweden), and an Anzai respiratory gating system, AZ-733VI (Anzai, Tokyo, Japan), including a laser sensor with visual guidance unit, ABLE (Anzai, Tokyo, Japan), where the gating was performed under abdominal compression. The Elekta Response (Elekta AB, Stockholm, Sweden) gating control interface was used to transfer the gating signal from the Anzai system to the Elekta linac, and the linac gun hold-on time was adjusted to the maximum allowable value of 6.50 sec to minimize the gate-on delay [11]. It was reported that the abdominal compression helped maintain the reproducibility of tumor motion trajectories during the computed tomography (CT) simulation, the pre-treatment tumor localization, and the beam delivery sessions [12]. To ensure accuracy, we configured the gating window for each patient by correlating the respiratory curve from the laser sensor and the tumor positions from the 4D CT images reconstructed with the aid of the respiratory curve. This allowed us to define a patient-specific gating window to keep the tumor displacement below 5 mm from the end-expiration, assuming the reproducibility of the tumor trajectories and the laser-based body surface measurements among the above three periods. This study was approved by the institutional review board of Tokyo Medical University with an approval ID of T2023-0199. Written informed consent was obtained from the patients involved in this study.

**Figure 1:**
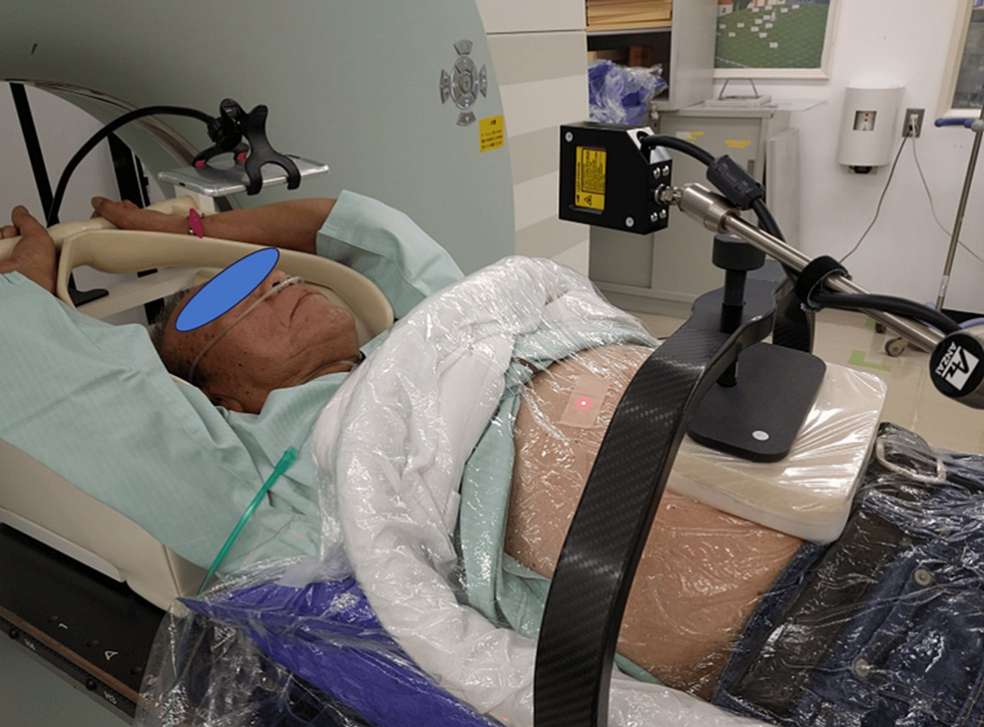
A photograph showing a CT simulation for lung tumor treatment planning under abdominal compression using a vacuum-assisted fixation device and an in-house compression block with a carbon fiber frame to apply pressure to the block. A narrow laser beam was projected perpendicular to the patient's abdomen with an aimed laser-abdomen distance of 12 cm. The laser spot was projected on the subcostal line midway between the inferiormost thoracic cage border and the L3 vertebral body to detect respiratory motion while minimizing the effect of the aortic pulsation. The reflected light was detected on a 1D array sensor, where the detected signal position indicates the distance to the abdomen. The laser sensor detects abdominal displacement caused by breathing and generates a respiratory curve, which was then exported to the CT unit to reconstruct multi-phase 4D CT images, where amplitude-based respiratory phase information was obtained from the respiratory curve. A portable display was placed over the patient's face to provide visual guidance for stable breathing. Treatment plans were created using the end-expiratory CT images. CT: computed tomography

Figure 1 shows a CT simulation for lung tumor treatment planning with a SOMATOM Emotion 16 (Siemens Healthineers AG, Erlangen, Germany) under abdominal compression using the vacuum-assisted fixation device, BodyFIX (Elekta AB, Stockholm, Sweden), and an in-house compression block made of Styrofoam (18 x 18 x 4 cm^3^) with a commercial carbon fiber frame, BodyFIX Diaphragm Control (Elekta AB, Stockholm, Sweden), to apply pressure to the block. A narrow laser beam was projected perpendicular to the patient's abdomen with an aimed laser-abdomen distance of 12 cm. The laser spot was projected on the subcostal line midway between the inferiormost thoracic cage border and the L3 vertebral body to detect respiratory motion while minimizing the effect of the aortic pulsation. The projected position was marked to accurately reproduce the measurement during the course of the treatment. The reflected light was detected on a one-dimensional array sensor, where the detected signal position indicates the distance to the abdomen. The laser sensor detects abdominal displacement caused by breathing and generates a respiratory curve, which was then exported to the CT unit to reconstruct multi-phase 4D CT images, where amplitude-based respiratory phase information was obtained from the respiratory curve. A surgical tape was applied to the laser spot on the BodyFIX vacuum cover sheet, thereby avoiding irregular reflection. A portable display included in the ABLE unit was placed over the patient's face to provide visual guidance for stable breathing as shown in Video 1. Treatment plans were created by Monaco 5.1.1 (Elekta AB, Stockholm, Sweden) treatment planning system (TPS) using the end-expiratory CT and the other two CT image sets giving 5 mm displacements before and after reaching the end-expiration. A gating window ITV was defined by these three CT image sets.

**Video 1:** Patient guidance display to help stabilize breathing. Breathing in and breathing out duration can be configured separately.

**Figure 2:**
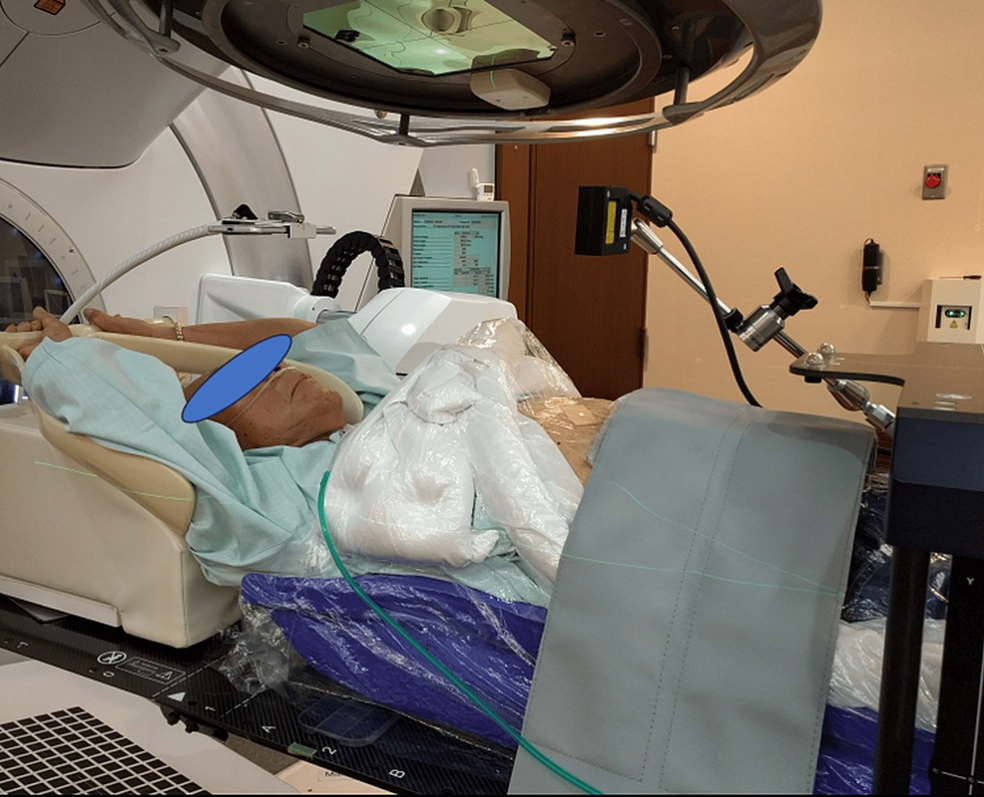
A photograph showing respiratory-gated lung tumor stereotactic radiotherapy under abdominal compression using the vacuum-assisted fixation device and the compression block with a 20 cm wide strap to apply pressure to the block. The laser sensor detects abdominal displacement caused by respiration and generates a respiratory curve. Then, gating thresholds were set on the respiratory curve by referring to the tumor displacement relative to the end-expiration, and the resulting gating signal was exported to a linac to control the beam delivery. Again, the portable display was placed over the patient’s face to visually guide stable breathing.

Figure 2 shows respiratory-gated lung tumor stereotactic radiotherapy under abdominal compression using the vacuum-assisted BodyFIX and the compression block with a 20 cm wide strap to apply pressure. The laser sensor detects abdominal displacement caused by respiration and generates a respiratory curve. Gating thresholds were set on the respiratory curve by referring to the tumor displacement relative to the end-expiration. The resulting gating signal was then exported to the linac to control the beam delivery. Again, the portable display was placed over the patient's face to visually guide stable breathing (Video 1). The carbon fiber frame used in the CT room was found to be too large for use with the linac, as its gantry head would hit the frame when the tumor was located far from the center of the body. The strap did not have this problem. The room-in to room-out for a patient required approximately 22 minutes including five minutes for fixation and setup, two minutes for pre-treatment CBCT imaging, one minute for pre-treatment couch adjustment, seven minutes for noncoplanar beam delivery with two minutes for couch rotation, two minutes for in-treatment gated CBCT imaging, and three minutes for release from the fixation. Besides, the first fraction required an additional minute for breathing practice.

**Figure 3:**
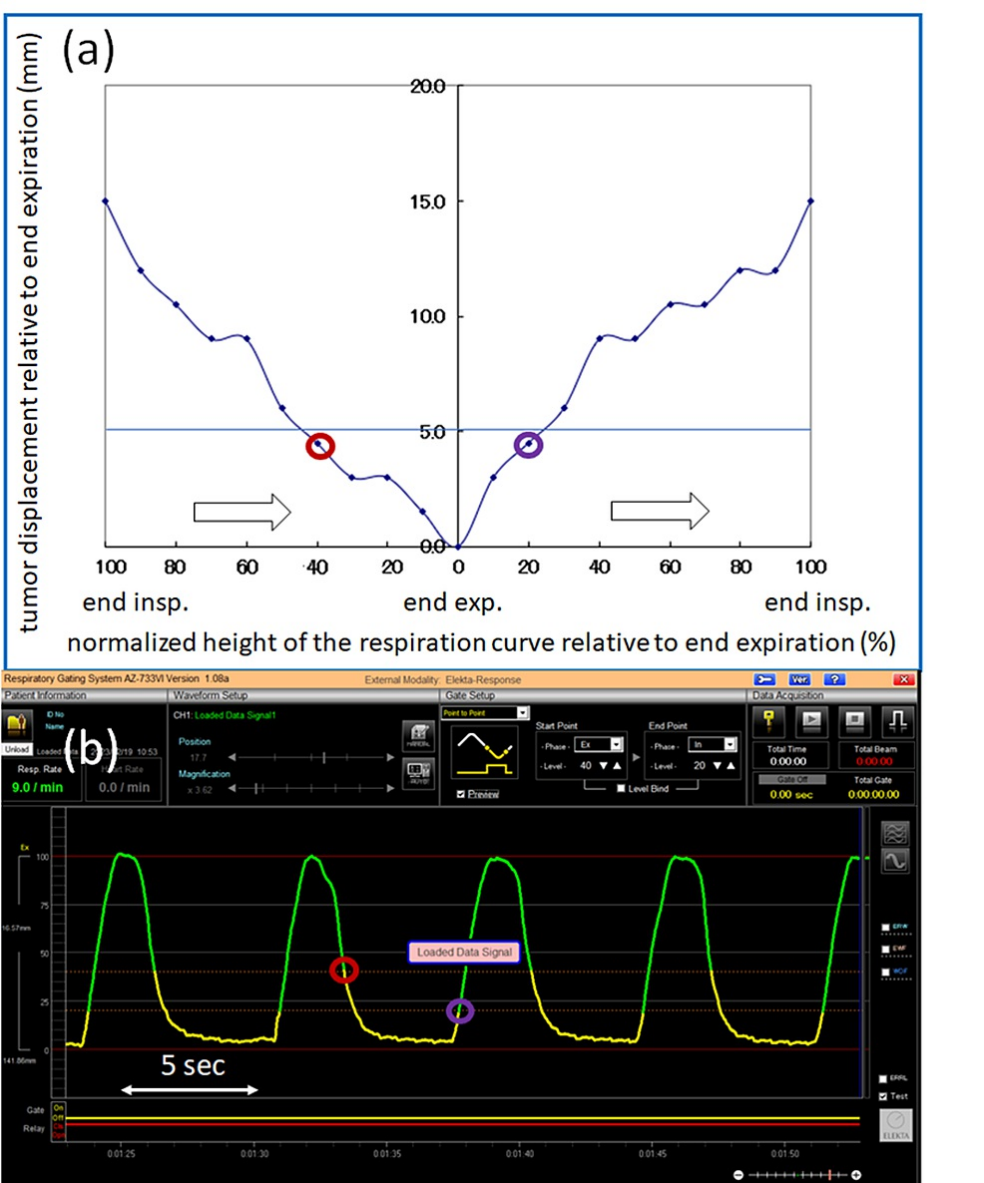
The tumor displacement in the superior-inferior direction and the corresponding respiration curve as a function of time. (a) A plot of the tumor displacement in the superior-inferior direction relative to end-expiration measured on 4D CT images as a function of the normalized height of the respiratory curve relative to end-expiration measured by the laser sensor, where the right half of the horizontal axis shows the respiratory amplitude moving from end-expiration (0%) to end-inspiration (100%) in every 10% step, while the left half shows the respiratory amplitude moving from end-inspiration (100%) to end-expiration (0%) in every 10% step. In this plot, 20-phase CT images were generated and utilized. The white arrows indicate the time course. (b) A corresponding respiration curve as a function of time, measured by the laser displacement sensor. The beam-on and beam-off thresholds were individually configured on the respiration curve by correlating the tumor displacement with the respiration curve. In other words, the thresholds were defined with respect to the tumor displacement plot in (a) and the respiratory amplitude plot in (b), where the tumor displacement was obtained relative to end-expiration. In these figures, the two thresholds were defined on the respiratory curve where the tumor displacement was 5 mm. The red and purple circles in (a) and (b) are the thresholds giving the tumor displacement of 5 mm relative to end-expiration and the gate-on periods are shown in yellow in (b). CT: computed tomography

Figure 3a depicts a plot of the tumor displacement in the superior-inferior direction relative to end-expiration measured on 4D CT images as a function of the normalized height of the respiratory curve relative to end-expiration measured by the laser sensor, where the right half of the horizontal axis shows the respiratory amplitude moving from end-expiration (0%) to end-inspiration (100%) in 10% increments, while the left half shows the respiratory amplitude moving from end-inspiration (100%) to end-expiration (0%) in 10% increments. In this plot, 20-phase CT images were generated and utilized. The time course is indicated by the white arrows. Figure 3b displays a corresponding respiration curve measured by the laser displacement sensor as a function of time. The beam-on and beam-off thresholds were configured on the respiratory curve by correlating the tumor displacement with the respiratory curve. The two thresholds were individually defined based on the tumor displacement in (a) and the respiratory amplitude in (b), relative to end-expiration. In these figures, the thresholds were set on the respiratory curve where the tumor displacement was 5 mm. The red and purple circles in (a) and (b) represent the corresponding thresholds giving the tumor displacement of 5 mm, and the gate-on periods are highlighted in yellow in (b).

**Figure 4:**
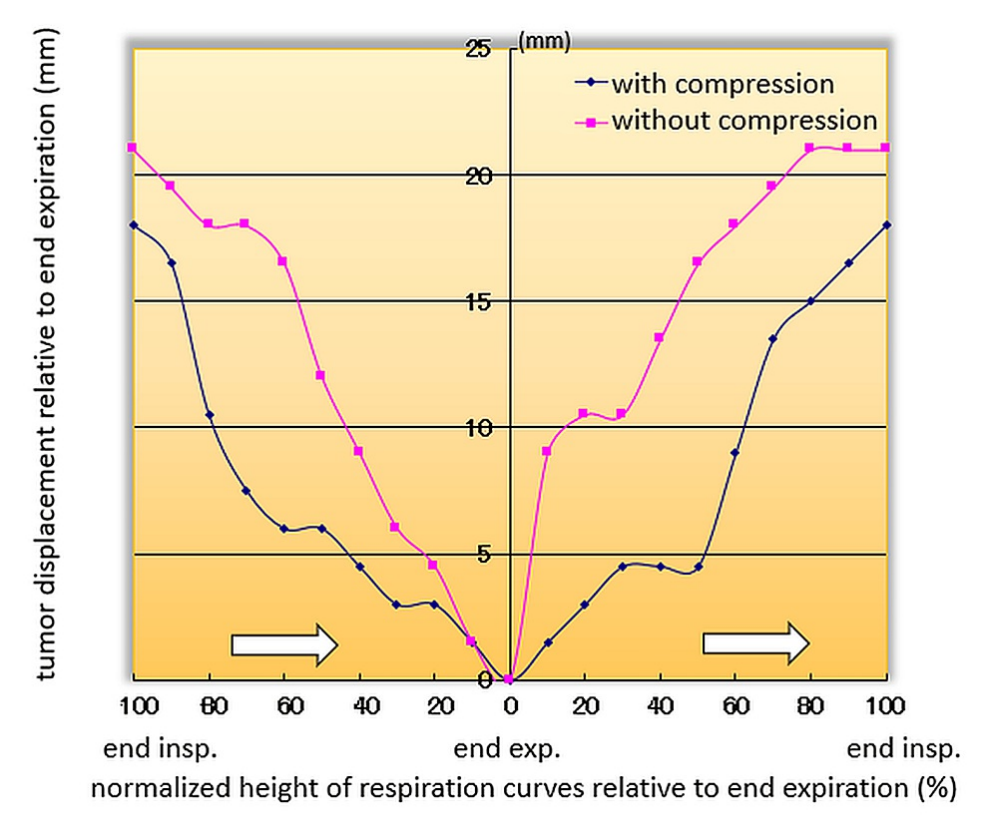
A comparison of tumor displacements relative to end-expiration for another patient with and without abdominal compression, as a function of the normalized height of the respiratory curves relative to end-expiration. The blue diamonds and red squares are with and without abdominal compression, respectively. The white arrows indicate the time course. If we allow the tumor displacement to be less than 5 mm relative to end-expiration for beam delivery, the abdominal compression increases the gating window from a range of (20%, 0%) to a range of (40%, 0%, 50%) in this particular case, resulting in a longer beam-on time per respiratory cycle.

Figure 4 compares tumor displacements relative to end-expiration for another patient with and without abdominal compression, as a function of the normalized height of respiratory curves relative to end-expiration. The blue diamonds and red squares represent displacements with and without abdominal compression, respectively. The white arrows indicate the time course. If the tumor displacement is allowed to be less than 5 mm relative to end-expiration during beam delivery, abdominal compression will increase the gating window from a 20-0% range to a 40-0-50% range in this particular case. This will result in a longer beam-on time per respiratory cycle.

Video 2 demonstrates the use of pre-treatment 4D CBCT imaging for daily registration of a lung tumor under abdominal compression. The patient couch was translated using the calculated registration errors. The video shows that after the couch adjustment, the tumor at end-expiration is observed at the superiormost position in the gating window ITV (in sky blue color), where the gating window ITV was defined as an ITV considering only respiratory phases within the gating window [13,14].

**Video 2:** Pre-treatment 4D CBCT imaging for daily registration of a lung tumor under abdominal compression. The patient couch was translated using the calculated registration errors. The video shows that the tumor at the end-expiration is observed at the superiormost position in the gating window ITV (in sky blue color) after the couch adjustment, where the gating window ITV was defined as an ITV considering only respiratory phases within the gating window. In other words, 4D CBCT imaging allows us to accurately and precisely position the tumor at the end-expiration, which is very important in our workflow. Respiratory hysteresis was also observed in the video, presumably leading to an unsymmetrical gating window as typically shown in Figures 3a, 3b. CBCT: cone-beam computed tomography; ITV: internal target volume

For further validation, intrafraction kV CBCT imaging was performed during gated-beam delivery. The kV projection images were acquired only when the measured respiratory signal amplitude corresponded to the preconfigured gate-on period, as typically shown in yellow in Figure 3b. The reconstructed CBCT images can be used to visualize the time-weighted tumor position during the gated-beam delivery.

**Figure 5:**
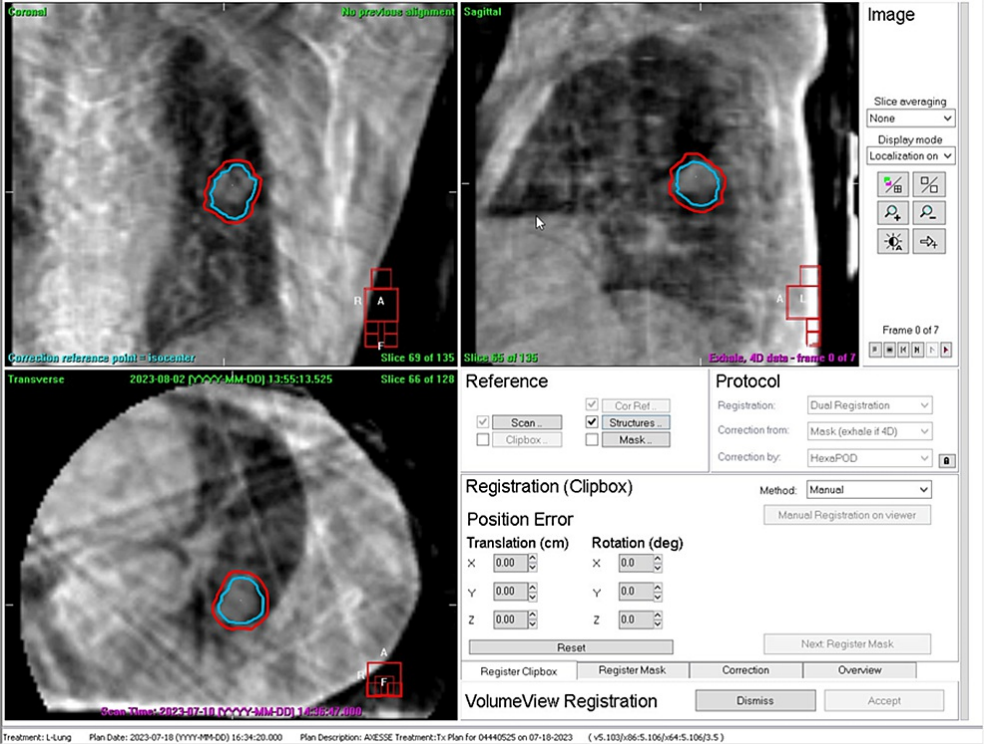
Respiratory-gated CBCT images acquired during stereotactic gated IMRT for a lung tumor case in Video 2 using a noncoplanar seven-port sliding-window technique with couch angles of 0°, 10°, and 350°. Specifically, the intrafraction half-scan CBCT images were acquired immediately after the first ports were completed with a 0° couch angle, with the linac gantry manually rotated 200° for CBCT imaging each time the measured respiratory signal amplitude met the preconfigured gate-on condition. During the CBCT data acquisition, the patient was instructed to maintain the same breathing pattern as before. In this figure, the gating window ITV is shown in sky blue and the planning target volume (PTV) is shown in red. The tumor appeared to remain within the gating window ITV during the stereotactic gated sliding-window IMRT. CBCT: cone-beam computed tomography; IMRT: intensity-modulated radiotherapy; ITV: internal target volume; PTV: planning target volume

Figure 5 displays the respiratory-gated CBCT images obtained during stereotactic gated intensity-modulated radiation therapy (IMRT) for a lung tumor case in Video 2. The noncoplanar seven-port sliding-window technique was used with couch angles of 0°, 10°, and 350°. Specifically, the intrafraction half-scan CBCT images were acquired immediately after completion of the first ports with a 0° couch angle, while the linac gantry was manually rotated 200° for CBCT imaging each time the measured respiratory signal amplitude met the preconfigured gate-on condition. The patient was instructed to maintain the same breathing pattern during this CBCT data acquisition. The figure displays the gating window ITV in sky blue and the planning target volume (PTV) in red, where the PTV was defined by adding an isotropic margin of 3 mm to the gating window ITV. The tumor appeared to remain within the gating window ITV during the stereotactic gated sliding-window IMRT.

To verify the delivered dose distribution, we created three-arc noncoplanar stereotactic flattening filter-free (FFF) volumetric modulated arc therapy (VMAT) plans and seven-port noncoplanar stereotactic sliding-window FFF IMRT plans with a photon energy of 6 MV using the Monaco TPS. These plans were created using the same CT image set of a lung tumor patient with a prescription dose of 50 Gy in five fractions. The plans were exported to the Elekta linac and the beams were delivered to the ArcCHECK quality assurance phantom (Sun Nuclear, Florida, USA). Global gamma pass rates were evaluated using a threshold of 10% and a criterion of 3 mm/3% in absolute dose mode.

Table 1 compares the global gamma pass rates and beam delivery times for non-gated and gated 6 MV FFF non-coplanar VMAT and IMRT deliveries using identical patient CT images and the dose prescriptions/constraints. The gamma pass rates (3 mm/3%) were 94% for VMAT and 99% for sliding-window IMRT cases. The beam delivery times were comparable between non-coplanar VMAT and non-coplanar sliding-window IMRT under either gated or non-gated conditions.

**Table 1:** Comparisons of global gamma pass rates and beam delivery times between non-gated and gated 6 MV FFF noncoplanar VMAT and IMRT deliveries using the identical patient’s CT images and dose prescriptions/constraints, where the global gamma pass rates were calculated with a threshold of 10% and a criterion of 3 mm/3% in absolute dose mode. The total MUs were also indicated. FFF: flattening filter-free; VMAT: volumetric modulated arc therapy; IMRT: intensity-modulated radiotherapy; CT: computed tomography; MU: monitor unit

	6 MV FFF VMAT (noncoplanar 3 arcs)		6 MV FFF sliding-window IMRT (noncoplanar 7 ports)	
	Non-gated	Gated	Non-gated	Gated
Global gamma pass rate (%)	94.4	94.3	99.2	99.2
Beam delivery time (sec)	133	373	140	375
Total MU	2377		2270	

## Discussion

We have clinically implemented gated stereotactic body radiotherapy under abdominal compression using the Anzai laser-based gating device with visual guidance in combination with our Elekta linac.

We showed that a patient-specific gating window with a prespecified maximum tumor displacement relative to the end-expiration can be obtained by acquiring a 4D CT consisting of 20 phase CT sets and a respiratory curve with the aid of the Anzai device. Using the Anzai device, respiratory hysteresis was also managed by setting two different thresholds on the respiratory curve based on the predetermined maximum tumor displacement relative to end-expiration. Without setting the two different thresholds, the gating window would not have been optimized for cases with respiratory hysteresis. 

Figure 4 showed that the abdominal compression minimized instantaneous tumor motion during 4D CT image acquisition, thereby leading to more stable respiratory management for the gating window setting. Another finding was that abdominal compression increased gating window width per cycle, thereby presumably leading to faster gated-beam delivery. To our knowledge, no studies have reported respiratory gating under abdominal compression on a standard linac. In addition, this is the first report suggesting that abdominal compression would accelerate the gated-beam delivery.

Video 2 shows that the 4D CBCT imaging allows us to accurately and precisely position the tumor at the end-expiration, which is very important in our workflow. Respiratory hysteresis was also observed in the video, presumably leading to an unsymmetrical gating window as typically shown in Figures 3a, 3b.

Figure 5 shows that the tumor appeared to remain within the gating window ITV during the stereotactic gated sliding-window IMRT, as the intrafraction gated CBCT provided the time-weighted tumor location during the gated IMRT delivery. This was made possible because the tumor position at the end-expiration was preadjusted immediately before beam delivery with the use of the 4D CBCT as shown in Video 2. To the best of our knowledge, no previous studies have reported the visualization of time-weighted tumor volume during intermittent delivery of gated IMRT beams.

Table 1 shows that the sliding-window gated IMRT had a better gamma index pass rate than the gated VMAT. Snyder reported that gated VMAT on an Elekta linac resulted in gantry overrun and rewind and showed reduced pass rates depending on the gantry speed [15]. The sliding-window gated IMRT does not have this issue; and therefore, our results appear to be consistent with Snyder's observation.

Limitations of this study include limited sample sizes, lack of comparative analyses against other respiratory management techniques, and potential institutional biases. Further studies with broader scope and larger sample sizes are necessary to further validate and refine the approach.

## Conclusions

We have described the clinical implementation of our respiratory-gated stereotactic body radiotherapy technique under abdominal compression using an Anzai laser-based gating device with visual guidance in combination with an Elekta linac. An initial validation result of the technique was demonstrated by acquiring intrafraction gated-CBCT images, showing that the tumor appeared to remain within the gating window ITV during the stereotactic gated sliding-window IMRT. More cases need to be evaluated to increase the validity.
